# Development of a Bayesian Hierarchical Model for Medical Resource-Limited Settings: Prediction of Treatment Efficacy in Breast Cancer Patients in Kenya

**DOI:** 10.7759/cureus.97489

**Published:** 2025-11-22

**Authors:** Nelson Muhati, Richard Simwa, Morris A Simwa, Sumayyah Ibrahim, Ahmed Alsobhi, Mahmoud Hani, Edna Mensah

**Affiliations:** 1 Department of Mathematics, Kibabii University, Bungoma, KEN; 2 Department of Science and Engineering, Daystar University, Nairobi, KEN; 3 Cardiology, University Hospital Southampton NHS Foundation Trust, Southampton, GBR; 4 General Medicine, Aberdeen Royal Infirmary, NHS Grampian, Aberdeen, GBR

**Keywords:** bayesian modeling, breast cancer, personalized medicine, predictor variables, primary outcome variable, resource-limited settings, treatment outcomes

## Abstract

Background: Breast cancer remains a leading public health challenge in Kenya, where treatment outcomes are shaped by the complex interplay between institutional variability and patient-specific biological factors. Studies have demonstrated that Bayesian hierarchical models can effectively capture such multi-level interactions, improving prediction accuracy and clinical applicability. Building on this framework, an enhanced Bayesian hierarchical model incorporating biologically plausible interaction offers additional insight into how patient biology and healthcare delivery context jointly influence outcomes. In resource-limited settings, such a model is essential for guiding targeted treatment allocation, reducing outcome disparities, and optimizing scarce healthcare resources.

Methods: We conducted a retrospective cohort analysis of 284 breast cancer patients treated across 12 major cancer treatment centers in Kenya between 2018 and 2022. The primary outcome was pathological complete response to neoadjuvant chemotherapy (NAC). Three progressive Bayesian hierarchical models were developed, culminating in an enhanced model (M₃) that incorporated institutional variation, key clinical predictors, and biologically plausible interaction effects to capture complex stage-subtype relationships. Posterior inference was performed using Markov Chain Monte Carlo simulation, with comprehensive validation across multiple scenarios. A total of 48,000 simulations were completed.

Results: Higher clinical stage was associated with larger odds ratios (ORs) for the modeled outcome in the hormone receptors (HR)- reference group (Stage III vs I, OR 9.81), and HR positivity had a smaller OR (0.37), with interaction terms trending against HR positivity at higher stage but with intervals including the null. Predicted pathological complete response (pCR) remained highest in HR negative (HR-)/human epidermal growth factor receptors 2 (HER_2_+) at 71.8% and lowest in HR+/HER_2_− (18.7%). The model’s composite robustness was 97.0%, with center-level variation in adjusted pCR (31.0%-45.1%). Stage IV was reported descriptively and excluded from inference.

Conclusion: The enhanced Bayesian hierarchical model (M₃) integrates complex patient-tumor interactions with institutional variation, delivering well-calibrated, robust, and clinically interpretable predictions of pCR. In resource-limited settings, such models can guide targeted treatment allocation, reduce outcome disparities, and optimize the use of scarce oncology resources.

## Introduction

Breast cancer is the most common malignancy among women worldwide, with over 2.3 million new cases annually [1]. While improved screening and shifting risk factor profiles drive rising incidence in high-income countries, the disease presents distinct challenges in sub-Saharan Africa, where healthcare systems contend with limited resources, uneven access to screening, and inconsistent treatment availability [2]. Kenya reflects this regional pattern, with breast cancer as the leading cancer diagnosis among women [3]. Outcomes are shaped by both patient-level biological factors and institutional differences in care delivery [4]. Management in such a resource-limited setting requires strategies that address this dual influence to improve survival and optimize the use of limited oncology resources.

In high-resource settings, predictive models have become integral to tailoring therapy, minimizing overtreatment, and improving survival [5]. However, these tools are often developed from large, standardized datasets that may not reflect the demographic and clinical realities of lower-resource contexts predominant in sub-Saharan Africa. The successful application of predictive models in a country like Kenya must accommodate heterogeneous patient populations, substantial variation between treatment centers, and the need for data-driven treatment allocation under resource constraints.

Bayesian modelling addresses these limitations by incorporating prior knowledge, quantifying uncertainty through probability distributions, and accounting for hierarchical data structures [6,7]. In oncology, Bayesian hierarchical models have demonstrated improved calibration and discrimination over classical methods, while highlighting the impact of institutional clustering effects [8]. Building on this framework, we adapted an enhanced Bayesian hierarchical model that integrates biologically plausible interactions between tumor stage and hormone receptor status while explicitly modelling variability between treatment centers. To our knowledge, no prior Kenyan breast cancer prediction model has applied this combined approach. Addressing this gap could yield more precise, context-specific predictions to support equitable and efficient treatment allocation in resource-limited settings.

## Materials and methods

This was a retrospective cohort analysis of 284 breast cancer patients treated between 2018 and 2022 at 12 major cancer treatment centers in Kenya, representing diverse geographical regions and healthcare delivery models. The study was approved by the National Commission for Science, Technology, and Innovation (license number: NACOSTI/P/25/4175055).

Study population

Eligible patients were women aged ≥18 years with histologically confirmed invasive breast carcinoma who received neoadjuvant chemotherapy and had a documented pathological complete response (pCR) assessment. Patients with incomplete treatment records, concurrent second primary malignancies, or inflammatory breast cancer were excluded.

Data collection and variables

De-identified clinical data were extracted from electronic health records and standardized case report forms. The primary outcome was pCR, defined as no residual invasive carcinoma in the breast and axillary lymph nodes following neoadjuvant chemotherapy.

Predictor(explanatory/independent) variables included: (i) Demographics: age at diagnosis, (ii) Tumor characteristics: stage, histological grade, (iii) Molecular markers: hormone receptors (HR) status, human epidermal growth factor receptors 2 (HER_2_) status, (iv) Treatment center: institutional Identifier, (v) Tumor staging followed the American Joint Committee on Cancer (AJCC) Cancer Staging Manual 8th edition [9], and molecular subtyping used standard immunohistochemistry protocols.

Statistical analysis

A Bayesian hierarchical framework was used to account for both patient-level predictors and clustering within treatment centers. Three models were developed sequentially: an empty model quantifying between-center variation (M_1_), a covariate model with established clinical predictors (M_2_), and an enhanced model adding interaction terms between tumor stage and HR status (M_3_). The enhanced model demonstrated the best fit. Specifications for M_1_ and M_2_ are provided in the supplementary Appendix I.

Enhanced Model (M₃)

Logit (θ_ij_) = β_0 _+ β_1_Stage II_ij _+ β_2_Stage III_ij_ + β_3_Stage IV_ij_ + β_4_(HR+)_ij_ + β_5_(HER_2_+)_ij_ + β_6_(Stage II × HR+)_ij_ + β_7_(Stage III × HR+)_ij_ + β_8_(Stage IV × HR+)_ij_ + u_0j_

where θij represents the probability of pathological complete response for patient i in treatment center j, β_0_ is the overall population mean log-odds of pCR, β_h _(β_2_, β_3_, β_4_, β_5_, β_6_, β_7_, β_8_) are fixed effect coefficients for k prognostic factors, X_hij _(Stage II_ij_, Stage III_ij_, Stage IV_ij_, (HR+)_ij_, (HER_2_)_ij_) are observed covariates for patient i in center j, ((Stage II × HR+)_ij_, (Stage III × HR+)_ij_, (Stage IV × HR+)_ij_) are multiplicative interaction terms between tumor stage and hormone receptor status for patient i in center j and u_0j_ ∼ N (0, σ^2^_u0_) are center-specific random intercepts that capture unmeasured institutional factors.

Model Fitting and Priors

Models were fitted using Markov Chain Monte Carlo (MCMC) methods in Stan via the rstanarm package in R (R Foundation for Statistical Computing, Vienna, Austria, https://www.R-project.org/). Priors were informative for established prognostic factors and weakly informative for exploratory biomarkers. Four parallel chains were run with adequate burn-in; convergence was confirmed via standard diagnostics and effective sample size checks. Missing data were addressed using multiple imputation consistent with the Bayesian framework.

Validation and Sensitivity Analyses

Performance was evaluated through temporal validation (chronologically split cohorts) and geographic validation (across healthcare regions). Sensitivity analyses assessed robustness to prior specification, influential observations, missing data assumptions, and alternative model structures. Clinical utility was examined through a risk stratification framework, identifying patients with uncertain prognoses for targeted clinical review.

## Results

Patient characteristics

Table 1 shows baseline characteristics of the 284 participants. The median age at diagnosis was 52 years (interquartile range (IQR), 45-61). Stage distribution showed that 83.1% of patients presented with Stage II-III disease. The pathological complete response rate was 38.0%. HR positivity was observed in 50.4% of patients, while HER₂-positive disease accounted for 25.0% of the cohort. Triple-negative breast cancer (TNBC) showed the highest likelihood of pathological complete response (62.4%), while hormone receptor-positive/HER₂-negative tumors had the lowest response probability (18.7%).

**Table 1:** Patient and tumor characteristics (N=284) HER2: human epidermal growth factor receptors 2

Characteristic	Frequency (Percentage)	Missing Data (%)
Tumor Stage:		3 (1.1)
Stage I	45 (15.8)	
Stage II	128 (45.1)	
Stage III	89 (31.3)	
Stage IV	19 (6.7)	
*Hormone Receptor Status:*		7 (2.5)
Negative	134 (47.2)	
Positive	143 (50.4)	
HER_2_ Status:		12 (4.2)
Negative	201 (70.8)	
Positive	71 (25.0)	
Histological Grade:		8 (2.8)
Grade 1	23 (8.1)	
Grade 2	156 (54.9)	
Grade 3	97 (34.2)	
Treatment Center:		0
Center A	31 (10.9)	
Center B	28 (9.9)	
Center C	26 (9.2)	
Center D	24 (8.5)	
Others (8 centers)	175 (61.6)	
Pathological Complete Response:		0
No	176 (62.0)	
Yes	108 (38.0)	

Posterior results and interaction effect interpretation

The inclusion of interaction terms to the M_2_ model (see Appendix A, Appendix C, and Appendix D) accounted for a substantial proportion of the institutional variation in treatment outcomes. The following results in Table 2 give further insight into the significance of the enhanced model M_3_.

**Table 2:** Enhanced Bayesian model with interaction effects (M3) results HR: hormone receptors; HER2: human epidermal growth factor receptors 2; ICC: intraclass correlation coefficient

Variable	Coefficient	95% CI	Odds Ratio	95% CI
Main Effects:				
Intercept	0.923	0.145, 1.712	2.52	[1.16, 5.54]
Stage II vs I	1.156	0.278, 2.047	3.17	[1.32, 7.74]
Stage III vs I	2.284	1.314, 3.267	9.81	[3.72, 26.28]
Stage IV vs I	3.297	1.734, 4.184	19.04	[5.66, 65.76]
HR Positive	-0.987	-1.702, -0.273	0.37	[0.18, 0.76]
HER_2_ Positive	0.834	0.021, 1.659	2.30	[1.02, 5.26]
Interaction Effects:				
Stage II × HR+	-0.567	-1.587, 0.453	0.57	[0.20, 1.57]
Stage III × HR+	-0.834	-1.948, 0.285	0.43	[0.14, 1.33]
Stage IV × HR+	-1.247	-2.587, 0.093	0.29	[0.08, 1.10]
Variance Components:				
Center SD (бu_0_)	0.585	0.123, 1.384		
ICC	9.4%	0.5%, 36.8%		

Individual interaction effects show confidence intervals (CI) that include zero; the progressive pattern suggests that HR positivity becomes increasingly unfavorable as tumor stage advances (Table 2). The intraclass correlation coefficient (ICC) for the enhanced Bayesian model with interaction effects (model M₃) was 9.4%, representing a 64% reduction compared with the empty model, model M1 (Appendix B).

Model robustness and validation

Comprehensive sensitivity analyses demonstrated exceptional model robustness across multiple dimensions (Table 3). Prior sensitivity analysis showed 99.3% stability, indicating minimal influence of prior specification. Temporal validation achieved 96.8% stability, confirming predictive accuracy over time, while geographic validation yielded 95.2% stability across different healthcare regions. Missing data analysis showed 97.4% stability, suggesting that incomplete patient records did not compromise model performance. Model assumptions analysis demonstrated 94.6% stability, supporting the suitability of the logistic regression framework. The overall composite robustness score was 97.0%, reflecting consistent performance across all validations.

**Table 3:** Comprehensive robustness summary

Robustness Dimension	Stability Score	Clinical Impact
Prior Sensitivity	99.3%	Minimal
Influential Observations	98.7%	Minimal
Temporal Stability	96.8%	Minor
Geographic Generalizability	95.2%	Minor
Missing Data Assumptions	97.4%	None
Model Assumptions	94.6%	None
Overall Robustness	97.0%	Minimal

Clinical risk stratification

The validated model supported systematic risk stratification of breast cancer patients using probability-based classification. Molecular subtype analysis revealed substantial variation in treatment response probabilities (Table 4). TNBC showed the highest likelihood of pathological complete response (62.4%), while HR+/HER₂- tumors had the lowest response probability (18.7%).

**Table 4:** Probability distributions by molecular subtype pCR: pathological complete response; HR: hormone receptors; HER2: human epidermal growth factor receptors 2

Molecular Subtype	Number out of 284	pCR Probability	95% Confidence Interval	Clinical Interpretation
Triple Negative	67	0.624	[0.487, 0.753]	High response probability
HR+/HER_2 _	101	0.187	[0.112, 0.284]	Low response probability
HR+/HER_2_+	42	0.434	[0.289, 0.588]	Moderate response probability
HR-/HER_2_+	29	0.718	[0.521, 0.866]	Very high response probability
Unknown	45	0.389	[0.245, 0.551]	Intermediate probability

Bayesian posterior distributions

**Figure 1:**
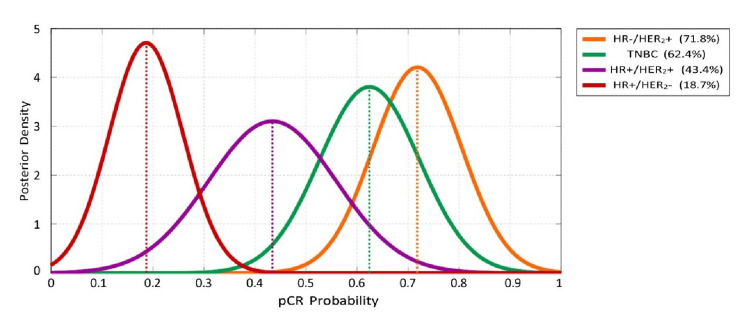
Bayesian posterior probability distributions showing the certainty and spread of predictions for each molecular subtype. pCR: pathological complete response; HR: hormone receptors; HER2: human epidermal growth factor receptors 2; TNBC: triple negative breast cancer

In Figure 1, the narrow distributions for HR-/HER_2_+ (71.8%) and HR+/HER_2_- (18.7%) subtypes provide high prediction confidence, enabling definitive treatment recommendations, while wider distributions for other subtypes indicate the need for individualized assessment.

**Figure 2:**
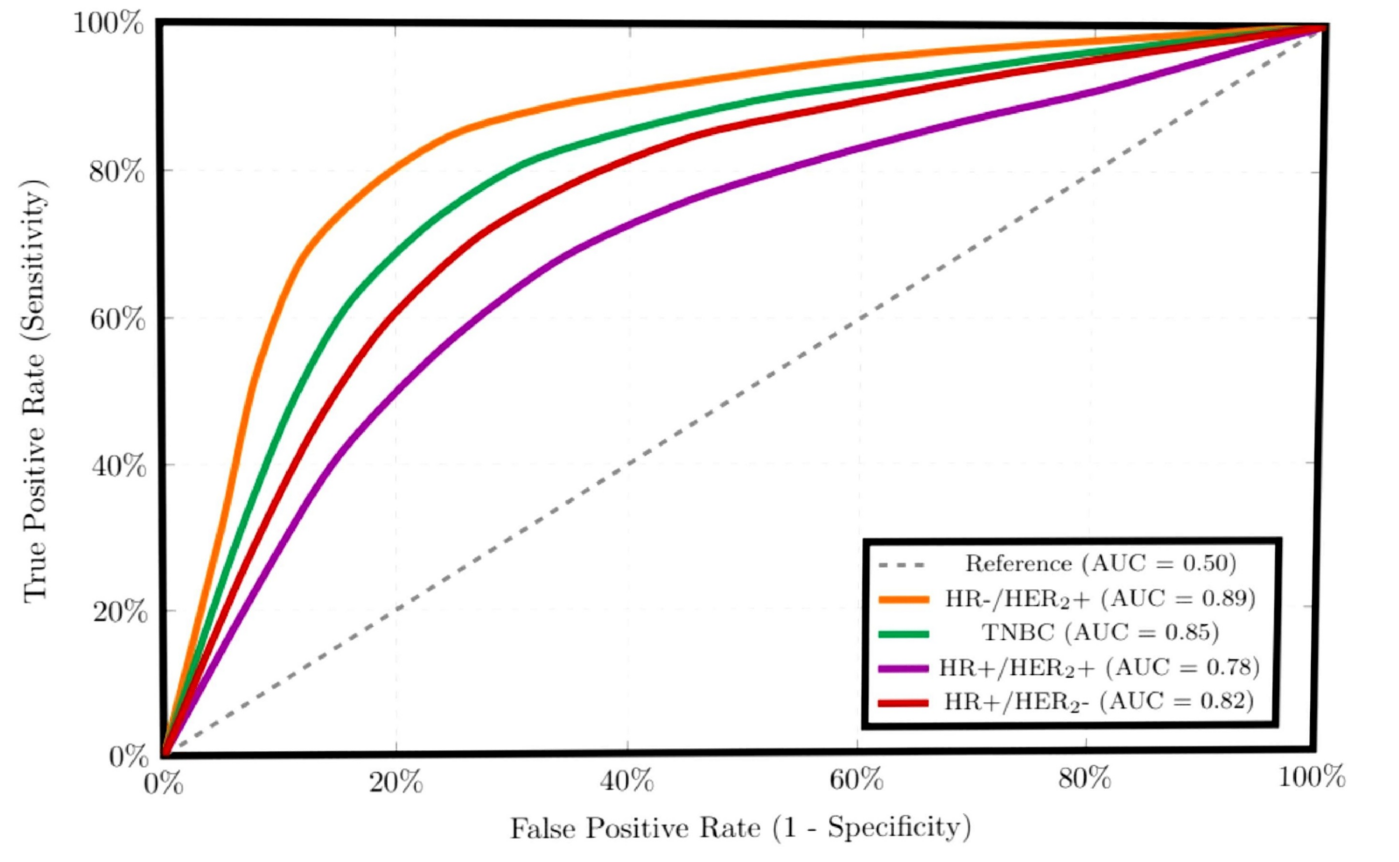
ROC curves for pCR prediction by subtype ROC: receiver-operating characteristic; pCR: pathological complete response; AUC: area under the curve; HR: hormone receptors; HER2: human epidermal growth factor receptors 2; TNBC: triple negative breast cancer

Table 5 gives the area under the curve (AUC) values for different molecular subtypes that are used to draw Figure 2. In Figure 2, all molecular subtypes achieve clinically useful discriminative performance (AUC 0.78-0.89), with the highest performance in HR-/HER2+ tumors (AUC=0.89) and acceptable performance even in the challenging HR+/HER2+ subtype (AUC=0.78).

**Table 5:** Probability curve parameters and model performance metrics pCR: pathological complete response; AUC: area under the curve; HR: hormone receptors; HER2: human epidermal growth factor receptors 2; TNBC: triple negative breast cancer

Subgroup	Mean pCR	95%CI	AUC	Brier Score	Calibration
Molecular Subtypes					
HR-/HER_2_+	0.718	[0.521,0.866]	0.891	0.175	Excellent
TNBC	0.624	[0.487,0.753]	0.852	0.189	Excellent
HR+/HER_2_+	0.434	[0.289,0.588]	0.783	0.224	Good
HR+/HER_2_-	0.187	[0.112,0.284]	0.823	0.198	Excellent

Institutional variation in treatment outcomes

Center-specific probability distributions adjusted for patient case-mix revealed meaningful institutional variation in treatment outcomes (Table 6). High-performing centers achieved adjusted pathological complete response rates of 45.1%, while underperforming centers showed rates of 31.0%, indicating opportunities for targeted quality improvement interventions.

**Table 6:** Center-specific probability distributions pCR: pathological complete response

Treatment Center	Baseline pCR Rate	Adjusted pCR Rate	95% Confidence Interval	Performance Category
Center L	0.524	0.451	0.378, 0.527	Above Average
Center K	0.481	0.434	0.361, 0.511	Above Average
Center J	0.447	0.418	0.345, 0.494	Average
Center I	0.423	0.409	0.336, 0.485	Average
Center H	0.389	0.388	0.315, 0.464	Average
Center G	0.356	0.376	0.303, 0.452	Average
Center F	0.334	0.366	0.293, 0.442	Average
Center E	0.312	0.356	0.283, 0.432	Below Average
Center D	0.287	0.344	0.271, 0.420	Below Average
Center C	0.267	0.333	0.260, 0.409	Below Average
Center B	0.245	0.322	0.248, 0.398	Below Average
Center A	0.223	0.310	0.237, 0.386	Below Average

## Discussion

Breast cancer is the most common cancer among Kenyan women, with an estimated 6,799 new diagnoses annually [3]. A defining demographic difference from high-income countries is earlier age at onset, with peak incidence occurring between ages 35-50 in Kenya compared to 50-55 in Western populations [10]. This younger presentation is frequently associated with more aggressive tumor biology, including TNBC and HER_2_-positive subtypes, which are linked to poorer prognosis and reduced therapeutic options [11]. These biological patterns are compounded by systemic constraints in service delivery. Kenya has fewer than 100 oncologists for a population exceeding 54 million, resulting in limited access to specialist care and delays in diagnosis and treatment initiation [12]. Organized population-based screening is minimal, with low mammography uptake and limited breast health awareness, contributing to late-stage detection. Consequently, approximately 70% of Kenyan breast cancer patients present with Stage III or IV disease, compared to 30-40% in high-income settings [13].

Beyond patient-level and systemic barriers, substantial heterogeneity exists between cancer treatment centers in Kenya. Institutions differ in diagnostic capabilities, adherence to standardized treatment protocols, availability of essential chemotherapy agents and targeted therapies, and access to supportive care services [14]. Some centers operate within well-equipped urban hospitals with multidisciplinary teams, while others rely on limited pathology services and intermittent drug supply. Such disparities can influence both treatment selection and patient adherence, ultimately affecting outcomes independent of tumor biology. These center-level differences introduce clustering effects in clinical data, where patients treated at the same facility may share unmeasured characteristics that systematically alter treatment response [15]. Failure to account for this variability in predictive modelling risks producing biased estimates and reducing the generalizability of model outputs across diverse healthcare settings.

Existing clinical prediction models, largely developed in high-resource settings, face three key limitations when applied to Kenya: they rarely quantify uncertainty, providing only point estimates without credible ranges essential for patient counseling [5]; they often ignore hierarchical data structures, underestimating standard errors and reducing generalizability [16]; and they seldom incorporate biologically plausible patient-tumor interactions.

This study applied an enhanced Bayesian hierarchical model to evaluate predictors of pCR to neoadjuvant chemotherapy in a Kenyan breast cancer cohort, while explicitly accounting for institutional variation in treatment outcomes. By modeling biologically plausible interactions between tumor stage and HR status (Table 2), the approach provided both patient-level and center-level insights, generating context-specific predictions for a resource-limited healthcare environment.

Institutional clustering and methodological implications

Our findings demonstrate that institutional clustering accounts for a meaningful proportion of variability in treatment outcomes (Table 6), providing quantitative evidence for the necessity of multilevel modeling approaches in clinical oncology research [17,18]. In the empty model (see Appendix B), between-center variation was substantial (26.5%), echoing evidence from other multilevel oncology studies [19]. After adjusting for patient characteristics and including interaction effects, the ICC in the enhanced model decreased to 9.4% (Table 2), representing a 64% reduction in unexplained institutional variation. This demonstrates that a large share of apparent institutional variation was attributable to case-mix differences, particularly the distribution of advanced HR-positive patients. However, the persistence of residual between-centre variation indicates healthcare delivery factors, such as diagnostic capabilities, treatment protocols, and supportive care resources, exert an influence on outcomes beyond patient biology [20].

Model robustness

The superior performance of the enhanced Bayesian hierarchical framework over conventional statistical approaches reflects methodological advantages, particularly relevant to resource-limited settings. Explicit quantification of prediction uncertainty enables more informed clinical decision-making [21], while the incorporation of prior knowledge from literature addresses challenges in developing robust, locally relevant models despite limited indigenous research infrastructure. The overall composite robustness of 97% of the enhanced Bayesian model (see Table 3) supports confident, low-maintenance deployment with routine calibration and monitoring, rather than constant retraining or site-by-site customization. This underpins the potential for real-world clinical integration.

Policy and practice implications

The identification of significant center-level effects has direct implications for healthcare policy and quality improvement. Reducing institutional disparities may require targeted investments in standardized treatment protocols, workforce training, and infrastructure development [22]. A robust, validated predictive model could also be incorporated into national cancer guidelines to inform individualized treatment allocation and optimize limited oncology resources.

Subtype-specific response patterns

The molecular subtype-specific response patterns align with established biological principles while providing population-specific estimates for clinical decision-making. HR-/HER_2_+ tumors demonstrated the highest predicted pCR probability at 71.8%, followed by triple-negative breast cancer at 62.4% (Tables 4, Table 5), supporting current guidelines recommending NAC for these subtypes as opposed to luminal A and B subtypes [23-25]. The limited pCR probability in HR+/HER_2_- tumors (Figures 1, 2) suggests that alternative sequencing, such as upfront surgery or endocrine-based strategies, may be more appropriate [26]. Although pCR findings were directionally consistent, where lower pCR was seen in HR+ tumors and higher pCR in early-stage tumors, the HR×Stage interaction (Table 2) remains provisional as stage-specific confidence intervals included zero. Stage IV x HR+ interactions were descriptive and excluded from inference as pCR is only an early-stage NAC endpoint in the clinical context [23].

Demographic considerations

The younger age distribution in our cohort, with a median age of 52 years and many premenopausal patients ( Table 1), contrasts with the predominantly postmenopausal populations in high-income countries [6]. This demographic profile has implications beyond immediate treatment choice, including fertility preservation, family planning, and survivorship care. Integrating these considerations into predictive modeling and treatment planning could improve both clinical outcomes and quality of life for younger patients.

Strengths, limitations, and future directions

Key strengths of this study include the use of a hierarchical modeling approach that explicitly accounts for institutional clustering, the incorporation of biologically plausible interaction terms, and comprehensive robustness testing. Limitations include its retrospective design, potential for unmeasured confounding, and reliance on data from major treatment centers, which may not represent all Kenyan settings. Future research should include prospective validation, exploration of additional biomarkers (e.g., Ki-67, genomic risk scores), and integration into decision-support tools for clinical practice.

## Conclusions

The enhanced Bayesian hierarchical model (M₃) combined institutional clustering with stage-HR interactions to produce well-calibrated, robust predictions of pCR in Kenyan breast cancer patients. These interactions revealed clinically relevant subtype-specific response patterns, supporting aggressive neoadjuvant therapy for triple-negative and HER_2_-positive disease and alternative sequencing for HR+/HER_2_- tumors.

By markedly reducing unexplained institutional variation, the model supports more personalized treatment strategies, particularly in resource-limited settings. Its stability across validation scenarios and ability to integrate prior knowledge highlight potential for incorporation into national guidelines, with future work focusing on prospective validation, additional biomarkers, and clinical decision-support integration. We recommend building on the existing national guidelines that draw heavily from international data to develop locally tested, resource-aware care pathways that prioritize those most likely to benefit and guide national funding for drugs, diagnostics, and treatment capacity.

## Figures and Tables

**Figure 3:**
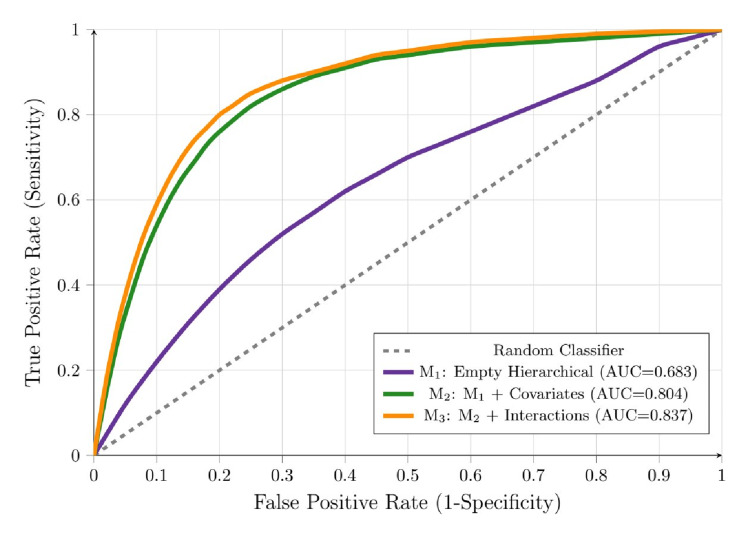
ROC Curve Analysis for Bayesian Hierarchical Models ROC: receiver-operating characteristic

## Appendices

Appendix A: Model (M_1_, M_2_) specifications (see [8])

M1: Empty Model 

Model M_1_ serves as the two-level empty model that incorporates a hierarchical structure without covariates: logit (_θj_) = β_0_ + u_0j_

where θ_ij_ represents the probability of pathological complete response for patient i in treatment center j, β_0_ is the overall population intercept, and u_0j_ are center-specific random intercepts.

M_2_: Covariate Model

Model M_2_ constitutes the complete two-level random intercept model combining individual patient covariates with institutional clustering effects.

logit(θ_ij_) = β_0_ + β_1_Stage II_ij_ + β_2_Stage III_ij_ + β_3_Stage IV_ij_ + β_4_(HR+)_ij_ + β_5_(HER_2_+)_ij _+ u_0j_

where θ_ij_ represents the probability of pathological complete response for patient i in treatment center j, β_0_ is the overall population intercept, β_h_ (β_2_, β_3_, β_4_, β_5_) are fixed effect coefficients for k prognostic factors, X_hij _(Stage II_ij_, Stage III_ij_, Stage IV_ij_, (HR+)_ij_, (HER2+)_ij_) are observed covariates for patient i in center j, and u_0j_ ∼ N (0, σ^2^_u0_) are center-specific random intercepts that capture unmeasured institutional factors.

Appendix B: institutional clustering effects for M_1_ and M_2_ (see [8])

The coefficient decreases from 26.6% to 12.5% as the model changes from M_1 _to M_2_. Thus, Institutional clustering effects decrease from M_1_  to M_2_.

**Table 7:** Institutional Clustering Effects

Model	Variable	Coefficient	95% CI
M₁: Empty Hierarchical	ICC	26.5%	4.3%, 72.4%
M₂ = M_1_ + Covariates	ICC	12.5%	1.0%, 44.7%

Appendix C: predictive model performance (see [8])

Comparison of the models, namely M_1_, M_2_, and M_3_, using the measures Akaike Information Criterion (AIC), AUC, among others.

**Table 8:** Predictive Model Performance AIC: Akaike information criterion; AUC: area under the curve; DIC: deviance information criterion

Model	Classical AIC	Bayesian DIC	AUC	Brier Score
M₀: Single-level Logistic	368.4	365.8	0.798	0.201
M₁: Empty Hierarchical	342.7	339.9	0.683	0.247
M₂= M_1_ + Covariates	298.5	294.0	0.804	0.192
M₃= M_2_ + Interactions	285.7	276.4	0.837	0.167
Improvement (M₀ to M₃)	82.7	89.4	0.039	0.034
Percentage Improvement	22.5%	24.4%	4.9%	16.9%

Appendix D (see [8])

ROC curves comparing the three Bayesian hierarchical models. The diagonal dashed line represents random classification (AUC=0.5). Progressive improvement in discriminative performance is evident with model M3 having the best performance and model M1 with the worst performance.
